# Exploratory Study on Th1 Epitope-Induced Protective Immunity against *Coxiella burnetii* Infection

**DOI:** 10.1371/journal.pone.0087206

**Published:** 2014-01-30

**Authors:** Xiaolu Xiong, Yong Qi, Jun Jiao, Wenping Gong, Changsong Duan, Bohai Wen

**Affiliations:** State Key Laboratory of Pathogen and Biosecurity, Beijing Institute of Microbiology and Epidemiology, Beijing, China; University of Texas Medical Branch, United States of America

## Abstract

*Coxiella burnetii* is a Gram-negative bacterium that causes Q fever in humans. In the present study, 131 candidate peptides were selected from the major immunodominant proteins (MIPs) of *C. burnetii* due to their high-affinity binding capacity for the MHC class II molecule H2 I-A^b^ based on bioinformatic analyses. Twenty-two of the candidate peptides with distinct MIP epitopes were well recognized by the IFN-γ recall responses of CD4^+^ T cells from mice immunized with parental proteins in an ELISPOT assay. In addition, 7 of the 22 peptides could efficiently induce CD4^+^ T cells from mice immunized with *C. burnetii* to rapidly proliferate and significantly increase IFN-γ production. Significantly higher levels of IL-2, IL-12p70, IFN-γ, and TNF-α were also detected in serum from mice immunized with a pool of the 7 peptides. Immunization with the pool of 7 peptides, but not the individual peptides, conferred a significant protection against *C. burnetii* infection in mice, suggesting that these Th1 peptides could work together to efficiently activate CD4^+^ T cells to produce the Th1-type immune response against *C. burnetii* infection. These observations could contribute to the rational design of molecular vaccines for Q fever.

## Introduction

*Coxiella burnetii* is an acidophilic, intracellular bacterium that causes acute and chronic Q fever in humans [1], [2]. The prevention of Q fever remains an important goal for both public health and international biosecurity [3]. An effective formalin-inactivated whole cell (phase I *C. burnetii*) vaccine has been in used in Australia for more than 20 years [4]. However, the vaccination can result in severe local or systemic adverse reactions, particularly when administered to populations that suffered previously from *C. burnetii* infection [5]. Consequently, investigators sought to combine immunogenic peptides with or without lipopolysaccharide (LPS) components to produce a vaccine that will not cause adverse reactions [3], [6], [7]. Chen *et al.* identified Th1-type T cell (Th1) epitopes in *C. burnetii* antigens that were targeted by antibody responses [8]. In addition, Zhang *et al.* demonstrated that a *C. burnetii* LPS-targeted B cell epitope conferred specific protection in the BALB/c murine model [9].

Previous studies suggested that both humoral and cellular immune responses are important for the host defense against *C. burnetii* infection [7], [10] and that cellular immunity, particularly that mediated by CD4^+^ T cells, is critical for this protection [8], [11]. The Th1 cytokines interferon (IFN)-γ and tumor necrosis factor (TNF)-α directly activate monocytes/macrophages and fibroblasts to control the intracellular growth of *C. burnetii* [12]–[14].

In this study, the CD4^+^ T cell epitopes of seven major immunodominant proteins (MIPs) of *C. burnetii* [15]–[17] were designed and synthesized based on online bioinformatic predictions to explore whether Th1 epitopes could confer an efficient protection against *C. burnetii* infection. IFN-γ secreting CD4^+^ T cells from mice immunized with *C. burnetii* were then used to screen the synthesized peptides. The Th1-positive epitopes selected by screening were then used to immunize mice for evaluation of their efficacies against *C. burnetii* infection.

## Materials and Methods

### Coxiella burnetii Strain

*Coxiella burnetii* Xinqiao strain (phase I) [18] was grown in embryonated eggs and purified by Renografin density centrifugation [19]. The purified *C. burnetii* organisms were extracted with trichloroacetic acid (TCA) to remove LPS, as described previously [20], and then suspended in phosphate-buffered saline buffer (PBS) (8.1 mM Na_2_HPO_4_, 1.9 mM NaH_2_PO_4_, 154 mM NaCl, pH 7.4) as whole cell antigens (WCA).

### Mice and Ethics Statement

Female C57BL/6J (B6) mice (6 weeks old) were purchased from the Laboratory Animal Center of Beijing in China. All the mice were maintained under biosafety level 3 conditions. The Laboratory Animal Administration Committee of Beijing pre-approved all animal experimental protocols, the ethical approval number was IACUC of AMMS-2013-008.

### Epitope Prediction of CD4^+^ T Cells

Seven MIPs including Com1 (CBU1910), GroEL (CBU1718), Mip (CBU0630), OmpA (CBU0307), OmpH (CBU0612), P1 (CBU0311), and YbgF (CBU0092) were scanned for 15-mer peptides predicted to have a high-affinity binding capacity for the MHC class II molecule H2 I-A^b^ (Table 1) using a consensus approach, as described previously [21]. Results were obtained using the ARB and SMM-align tools on the IEDB website, and all peptides were ranked according to their predicted affinity by each method. The predicted peptides with the highest median ranks were then selected and synthesized. A set of 131 different peptides was synthesized by SBS Genetech Co. (Beijing, China) as coarse purity materials (>70% purity) that were used in the initial peptide screening. Peptides used in flow cytometry and immunization experiments were re-synthesized as high quality pure materials (>98% purity).

**Table 1:** Summary of antigen selection and Th1 epitope prediction.

COGs and ProteinAnnotation	Genename	Locus tag	Signalpeptide^a^(SignalP/LipoP)	SubcellularLocation^b^(PSORTb/SOSUIGramN)	Proteinlength (aa)	Number of predicted I-Abbinding peptides
27 kDa outer membraneprotein	*com1*	CBU_1910	Yes/SpI	Unknown/PP	245	2
Chaperonin GroEL	*groEL*	CBU_1718	No/No	C/C	552	26
Peptidyl-prolyl cis-transisomerase Mip	*mip*	CBU_0630	Yes/SpI	OM/OM	230	15
OmpA-like transmembranedomain protein	*ompA*	CBU_0307	Yes/SpI	OM/OM	231	17
Putative outermembrane skp	*ompH*	CBU_0612	Yes/SpI	PP/OM	165	15
outer membraneprotein P1	*p1*	CBU_0311	Yes/SpI	Unknown/EC	252	29
tol-pal systemprotein YbgF	*ybgF*	CBU_0092	Yes/SpI	Unknown/OM	305	17

a Signal peptides and signal peptide type of each protein were predicted with software SignalP 4.0 or LipoP 1.0, which are available online (http://www.cbs.dtu.dk/services/SignalP-4.0 and http://www.cbs.dtu.dk/services/LipoP-1.0. Accessed 30 March 2013).

b The subcellular localization of each protein was predicted using PSORTb 3.0.2 or SOSUI-GramN, which is available online (http://www.psort.org/psortb/index.htmland http://bp.nuap.nagoya-u.ac.jp/sosui/sosuigramn/sosuigramn_submit.html. Accessed 22 January 2013).

SpI, signal peptide (signal peptidase I); EC, extracellular; OM, outer membrane; PP: periplasmic; C, cytoplasmic.

### Preparation of Recombinant Proteins

The genes encoding MIPs from *C. burnetii* were amplified with corresponding primer pairs by PCR, and cloned into the plasmid pET32a (Novagen, Madison, WI) or pQE30 (Qiagen GmbH, Hilden, Germany) [16]. Recombinant MIPs were expressed as 6×His-tagged fusion proteins in *Escherichia coli* BL21 (Novagen) or M15 (Qiagen GmbH), and purified using Ni-NTA agarose (Qiagen GmbH) as described previously [16].

### IFN-γ Recall Responses in CD4^+^ T Cells Assayed by ELISPOT

Five mice per group were immunized subcutaneously (s.c.) with 20 µg recombinant protein or WCA of *C. burnetii* in the context of complete Freund’s Adjuvants (CFA, Sigma-Aldrich, St. Louis, MO), and sacrificed on day 10 post-immunization. Lymph nodes and spleens were harvested from immunized mice and homogenized in to cell suspensions, from which CD4^+^ T cells were isolated using CD4^+^ magnetic micro-beads (Miltenyi, Auburn, CA). Antigen-specific IFN-γ recall responses were measured in the purified CD4^+^ T cells by ELISPOT, as described previously [8]. Mononuclear cells were isolated from naïve mouse spleens as antigen-presenting cells (APC) [22]. Approximately 2×10^5^ purified CD4^+^ T cells were incubated with 1×10^5^ APC in 100 µl complete 1640 medium (Hyclone, Beijing, China) containing 10% (vol/vol) fetal bovine serum (FBS, Hyclone) in each well of a 96-well ELISPOT plate (Mabtech AB, Nacka Strand, Sweden). Two micrograms of each cognate peptide was then added to triplicate wells, and incubated for 20 h at 37°C. An AT-Spot 3000 ELISPOT reader (Antai Yongxin Medical Technology, Beijing, China) was then used to count the number of spot forming cells (SFC) following peptide stimulation. For each peptide, the stimulation index (SI) was calculated using the number of SFC in peptide-stimulated cells divided by the number of SFC in media-stimulated cells, and a SI >2 was considered positive [8].

### Intracellular Cytokine Staining (ICCS) and Flow Cytometry Analysis

Approximately 2×10^6^ splenocytes (pooled from 5 mice immunized with *C. burnetii* WCA) were incubated with 10 µg of each peptide or WCA in a total volume of l ml complete 1640 medium containing 10% FBS in each well for 18 h at 37°C. Next, 1.4 µl Golgistop was added to each well, and plates were incubated for another 6 h. Splenocytes were then harvested, washed with 1 ml permeabilization buffer, and stained with anti-CD3e-PerCP, anti-CD4-APC, anti-IFN-γ-PE, or anti-TNF-α-FITC antibodies for 15 min at room temperature. For the positive control, 2×10^6^ splenocytes were incubated with 1 ml of fresh medium containing 1 µg/ml Phorbol myfismte acetate, 100 ng/ml ionomycin, and 1.4 µl of Golgistop for 4 h, after which cells were stained as described above. All reagents used in this assay were purchased from BD Pharmingen (San Jose, CA).

### Proliferation Assay in CD4^+^ T Cells

Approximately 4×10^5^ CD4^+^ T cells from mice immunized with *C. burnetii* WCA were incubated with 2×10^5^ APCs in a total volume of 1 ml complete 1640 medium containing 10% FBS in each well of a 24-well plate (Corning, Corning, NY). Ten micrograms of each peptide was then added to triplicate wells and incubated for 48 h at 37°C. Finally, 10 µl CCK-8 reagent (Dojindo, Shanghai, China) was added to each well for 4 h, and the absorbance at 450 nm (OD_450_) was measured using a UVM340 enzyme symbolized meter (Biochrom, Berlin, Germany). The background OD_450_ from culture medium without cells was subtracted from each experimental value. The proliferation of antigen-specific CD4^+^ T cells was calculated as the absorbance of T cells stimulated with each peptide divided by T cells stimulated with PBS alone.

### Adoptive Transfer of CD4^+^ T Cells

Lymph nodes and spleens were collected from 5 mice immunized s.c. with *C. burnetii* WCA (20 µg/per mouse) on day 28 after immunization. CD4^+^ T cells were isolated from the lymph nodes and spleens using CD4^+^ magnetic micro-beads [23]. Six mice per group were adaptively transferred with 10^7^ CD4^+^ T cells via *tail vein* injection. Twenty-four hours later, each mouse was challenged with 10^6^ *C. burnetii* by intraperitoneal injection (i.p.). On day 7 after challenge, the mice were sacrificed, their spleens were harvested and weighed, *C. burnetii* loads were evaluated using quantitative real-time PCR (qPCR) specific for *C. burnetii* [23].

### Mouse Immunization with Peptides

Fifteen mice were immunized with 20 µg of each peptide or a pool of 7 peptides in the context of CFA on day 0, followed by 20 µg cognate peptides in the context of incomplete Freund’s Adjuvants (IFA, Sigma-Aldrich, St. Louis, MO) on days 14 and 28 after the primary immunization. In parallel, fifteen mice were immunized with 20 µg of *C. burnetii* WCA or PBS mixed with CFA or IFA as positive and negative controls, respectively.

### Analyses of Antibodies and Cytokines in the Sera of Peptide-immunized Mice

Three mice from each immunization group were sacrificed on day 14 after treatment, and serum were separated from whole blood. Antibodies against *C. burnetii* WCA, recombinant proteins, or peptides were measured by ELISA as described previously [24]. Briefly, 100 µl of antigen solution (2 µg/ml) was applied to each well to coat the microplates (Corning, Corning, NY), and each serum sample was diluted 1∶50. The optical density (OD) of each well was read at 450 nm using a UVM340 microplate reader. A multiplex immunoassay in a Luminex Bio-Plex 200 IS 100 instrument (BIO-RAD, Hercules, CA) was also used to quantify serum cytokines including interleukin-2 (IL-2), interleukin-4 (IL-4), interleukin-10 (IL-10), interleukin-12p70 (IL-12p70), IFN-γ, and TNF-α. All measurements were performed in at least triplicate, as described previously [24], [25]. Multiplex kits and related reagents were purchased from Affymetrix (Santa Clara, CA).

### Immunized Mice Challenged with *C. burnetii*

Six mice per immunization group were challenged i.p. with 10^6^ *C. burnetii* on day 14 after the third immunization. Seven days after challenge, mice were sacrificed, and their spleens were harvested for measurement of splenomegaly and quantification of *C. burnetii* DNA using qPCR [23].

### Statistical Analysis

SFC, intracellular cytokines, T cell proliferation, spleen weights and *C. burnetii* load were compared between groups using one-way analysis of variance (ANOVA) followed by Student-Newman-Keuls (SNK) test using SAS 9.1 software (SAS Institute Inc., Cary, NC). The levels of serum antibodies and cytokines were analyzed by ANOVA for a 2-way factorial design using SAS 9.1 software. *P* values of <0.05 were considered to be significant.

## Results

### Screening of Th1 Epitopes in the Major Immunodominant Proteins by ELISPOT Assay

Using consensus analysis, 131 peptides from 7 MIPs of *C. burnetii* were predicted to have high-affinity binding capacity to H2 I-A^b^, including 2 epitope peptides identified previously from Com1 [8] that were used as positive controls. Thirty-four out of these 131 candidate peptides elicited positive responses in CD4^+^ T cells from mice immunized with parental proteins (Table S1), whereas no peptides elicited a positive response in CD4^+^ T cells isolated from naïve mice. Nine pairs of the 34 peptides overlapped by 11 or more residues. Specifically, seven pairs contained two overlapping peptides, and two pairs contained three, giving a total of 20 overlapping peptides. The peptides with the highest SI values were selected as the representative peptide of each pair.

### Identification of Th1 Epitopes with CD4^+^T Cells from *C. burnetii* Immunized Mice

The peptides identified during the initial screening were tested for recognition by CD4^+^ T cells isolated from 5 mice immunized with *C. burnetii* WCA. Seven of these 22 peptides stimulated significantly increased levels of IFN-γ secretion in CD4^+^ T cells from WCA-immunized mice compared with mock stimulation. The 7 distinct epitopes were identified in 7 different *C. burnetii* MIPs (Table 2). BLAST searches of the amino acid sequences using the National Center for Biotechnology Information (NCBI) web pages and databases revealed that the Th1 epitope sequences were highly conserved among *C. burnetii* strains, and were different from the sequences found in other bacterial species.

**Table 2:** Summary of seven distinct Th1 epitopes identified by ELISPOT and MTT assay.

Identified peptide ID	Sequence	IFN-γ T cell ELISPOT [SI]^a^		CD4^+^ T cell proliferation^d^
		SI^a^-rAg^b^	SI^a^-WCA	
			immunization^c^	
Com1_42–56_	HYLVNHPEVLVEASQ	9.17	24.83	1.34
GroEL_474–488_	DVNYGYNAATGEYGD	6.25	24.62	1.27
Mip_159–173_	FDSSYKRGQPATFPL	2.1	12.41	1.32
OmpA_146–160_	GKLGVAYTYNRANAG	2.78	4	1.29
OmpH_13–27_	VAMIWSVAAVAQTVG	2.82	4	1.4
P1_70–84_	PVSASITQFGPVGEL	2.23	16	1.4
YbgF_185–199_	LLTKKQYDKAQASFQ	2.14	9.33	1.27

a Stimulation index (SI) was calculated using the number of SFC in peptide-stimulated cells divided by the number of SFC in media-stimulated cells, and a SI >2 was considered to be positive.

b 2×10^5^ CD4^+^T cells were isolated from 5 mice immunized with 20 µg recombinant protein on day 10 post immunization. Each peptide was added at a concentration of 2 µg/ml for 20 h. The data presented are the mean of three independent experiments.

c 2×10^5^ CD4^+^ T cells were isolated from 5 mice immunized with 20 µg *C. burnetii* WCA on day 10 post-immunization. Each peptide was added at a concentration of 2 µg/ml for 20 h. The data presented are the mean of three independent experiments.

d 4×10^5^ CD4^+^T cells were isolated from 5 mice immunized with 20 µg *C. burnetii* WCA on day 10 post immunization. Each peptide was added at a concentration of 10 µg/ml for 48 h. The proliferation levels of CD4^+^ T cells are the mean of three independent experiments.

### IFN-γ and TNF-α Production of CD4^+^ T Cells from *C. burnetii* Immunized Mice

**Figure 1:**
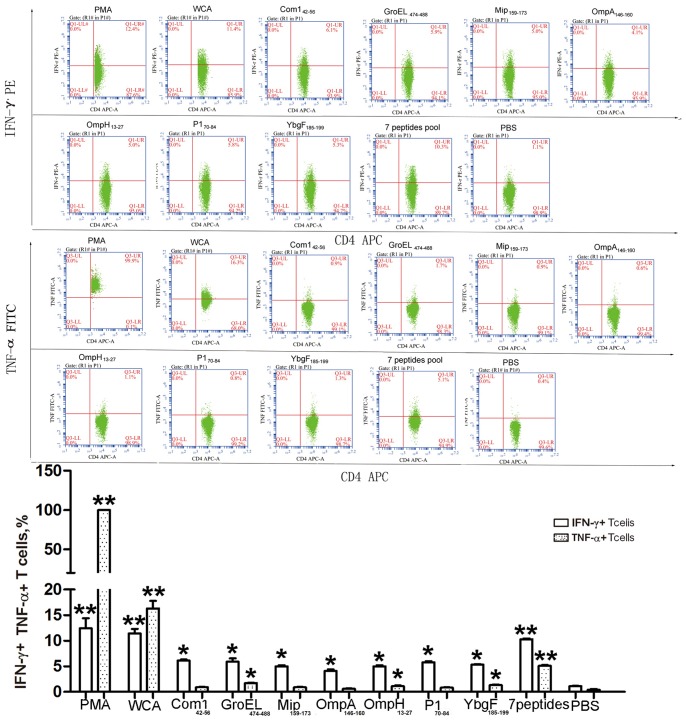
Epitope peptide-induced IFN-γ and TNF-α expression in CD4^+^ T cells. After 24-h stimulation with peptides, the expression of IFN-γ/TNF-α in CD4^+^ T cells isolated from *C. burnetii* WCA-immunized mice was measured by flow cytometry. T cells cultured with PBS served as negative controls. The data are representative of three independent experiments, and the percentages of double positive cells in the T cells are indicated in the top right corners. The means and standard deviations from the results of three independent experiments are also shown. Compared with PBS stimulation; *P<0.05; **P<0.01.

Seven peptides were used individually or pooled to stimulate CD4^+^ T cells from *C. burnetii* WCA-immunized mice, and then the levels of the Th1-type cytokines IFN-γ and TNF-α were analyzed by flow cytometry. Compared with mock stimulation, each of the 7 peptides induced significantly higher levels of IFN-γ, and three stimulated significantly higher levels of both IFN-γ and TNF-α (Figure 1). Furthermore, treatment with the pooled 7 peptides resulted in levels of IFN-γ and TNF-α that were increased significantly compared with any individual peptide. The IFN-γ levels induced by the pooled peptides were comparable to that induced by *C. burnetii* WCA, whereas the induced TNF-α levels were only one third of that induced by WCA.

### Proliferation of CD4^+^ T Cells Isolated from *C. burnetii* Immunized Mice

Twenty-two peptides identified in the initial screening were incubated individually with CD4^+^ T cells isolated from mice immunized with *C. burnetii* WCA to assess their ability to stimulate T cell proliferation. Compared with PBS, 10 of the 22 peptides induced significantly higher absorbance values (corresponding to proliferation) in CD4^+^ T cells (Table S1).

### Measuring Antibodies and Cytokines in the Serum of Peptides Immunized Mice

**Figure 2:**
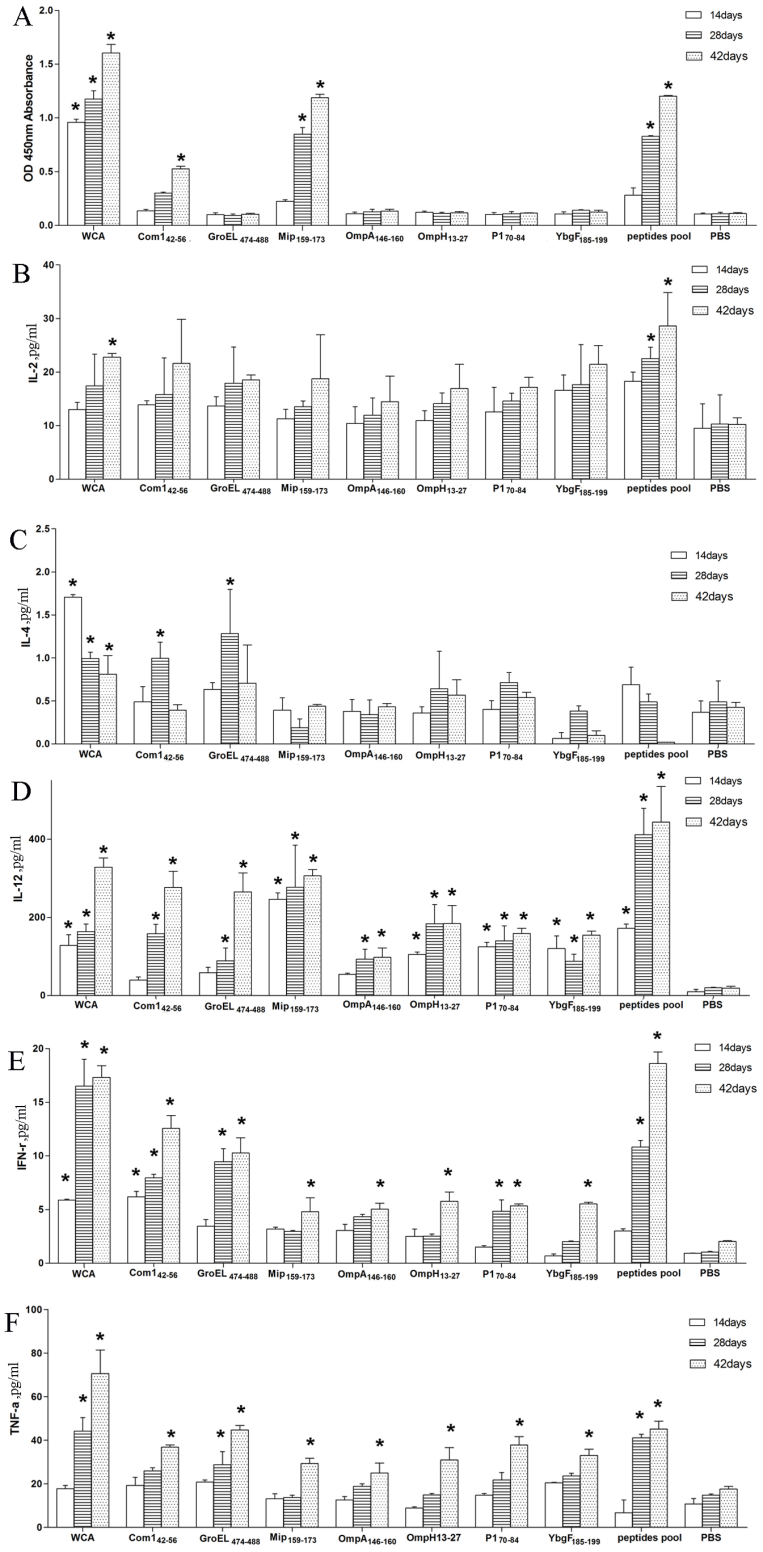
Measurement of antibodies and cytokines in the serum of immunized mice. Mice were immunized with 7 peptides individually or pooled three times at 2 week intervals. Mice immunized with WCA or PBS served as positive and negative controls, respectively. Serum samples were collected from 3 mice per group 14 days after each immunization, and antibodies against WCA, and parental proteins or peptides were measured by ELISA, and the cytokines IL-2, IL-4, IL-10, IL-12p70, IFN-γ and TNF-α were determined using Luminex technology. Compared with PBS immunization at the same time; *P<0.05.

Mice were immunized three times with the individual or pooled 7 peptides, and sera were collected on day 14 after each immunization. Antibodies against *C. burnetii* or parental recombinant proteins were not detectable in the sera of mice immunized with any peptide or the peptide pool when assessed by ELISA. Only sera from mice immunized with peptide Com1_42–56_, Mip_159–173_ or pooled 7 peptides displayed detectable antibodies against the cognate peptides (Figure 2).

Luminex assays revealed that the levels of IL-2, IL-12p70, IFN-γ, and TNF-α, but not IL-4, were significantly higher in sera from mice immunized with peptide pool compared with PBS-immunized mice at 28 or 42 days post-immunization. In addition, IL-10 was undetectable in all serum samples.

### Protection against *C. burnetii* in CD4^+^ T Cells after Adoptive Transfer

**Figure 3:**
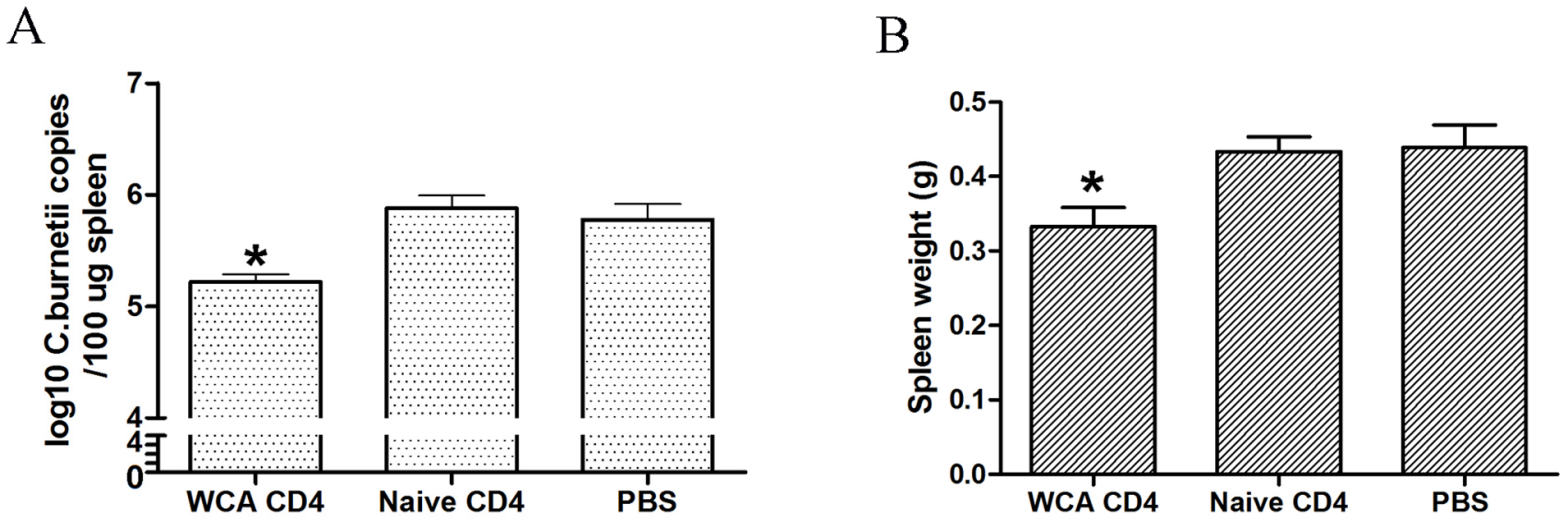
Immunoprotection against *C. burnetii* induced by the adoptive transfer of Coxiella-specific CD4^+^ T cells. CD4^+^ T cells from *C. burnetii* WCA-immunized mice were transferred to groups of six naïve mice, and each mouse was challenged with *C. burnetii* 24 h post-transfer. On day 7 after challenge, the mice were sacrificed and their spleens were harvested for the detection of *C. burnetii* DNA by Qpcr (A) and measurement of spleen weights (B). Data are expressed as the mean of 6 mice, and error bars indicate the standard deviation. Compared with the negative control; *P<0.05.

CD4^+^ T cells isolated from *C. burnetii* WCA-immunized mice were transferred to naïve mice. Seven days after challenge with *C. burnetii*, the bacterial load in the spleens of mice receiving CD4^+^ T cells from WCA-immunized mice were significantly lower than those receiving CD4^+^ T cells from naïve mice (Figure 3A). The spleen weights of mice receiving CD4^+^ T cells from WCA-immunized mice were also significantly lighter than those receiving CD4^+^ T cells from naïve mice (Figure 3B).

### Protection against *C. burnetii* Infection Induced by Epitope Peptides

**Figure 4:**
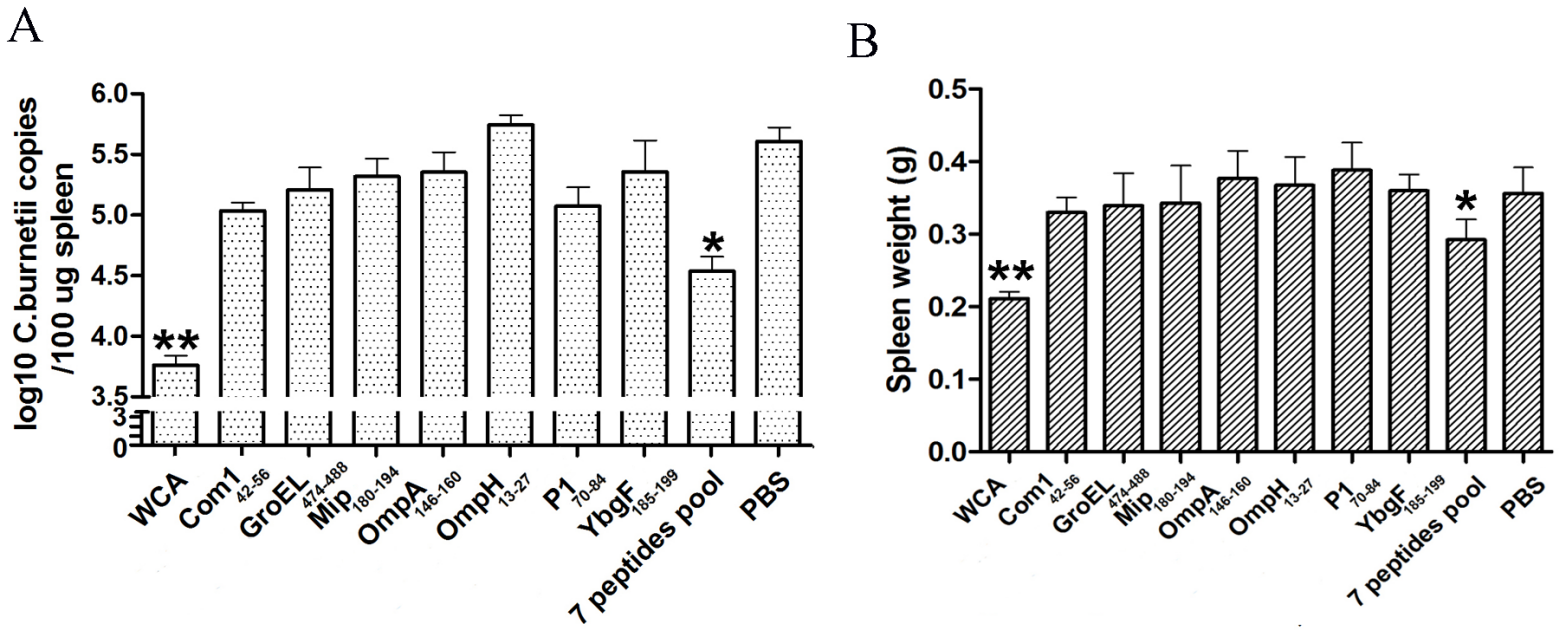
Evaluation of the protective efficacy of peptides by analysis of the Coxiella burden in immunized mice. Six naïve mice per group were immunized with 7 peptides individually or pooled. Mice immunized with *C. burnetii* WCA and PBS served as positive and negative controls, respectively. Fourteen days after the last immunization, each mouse was challenged with *C. burnetii*. The mice were sacrificed on day 7 after challenge, Coxiella loads in the mouse spleens were determined by Qpcr (A) and spleen weights were measured (B). Data are expressed as the mean copy number of six mice, and error bars indicate the standard deviation. Compared with the negative control; *P<0.05.

Mice immunized with the seven individual peptides or the pooled 7 peptides were challenged with *C. burnetii*. Both the bacterial loads and spleen weights of mice immunized with WCA or the peptide pool were significantly lower than mice immunized with PBS (Figure 4A, B). However, no significant difference in bacterial loads and spleen weights was detected between mice immunized with any individual peptide and mice immunized with PBS. In addition, the bacterial loads and spleen weights of mice immunized with *C. burnetii* WCA were significantly lower than mice immunized with the peptide pool.

## Discussion

The T cell-mediated immune response to *C. burnetii* infection is well studied. Zhang *et al* demonstrated that the adoptive transfer of splenocytes from *C. burnetii* WCA-immunized mice could protect against *C. burnetii* infection in mice [10]. Subsequently, Read *et al* demonstrated that SCID mice injected with CD4^+^ T cells alone were able to control the primary *C. burnetii* infection [26]. However, whether the antigen-specific CD4^+^ T cells isolated from *C. burnetii*-immunized mice could protect against *C. burnetii* infection remains unclear. In this study, we demonstrated that CD4^+^ T cells from mice immunized with LPS-removed WCA from *C. burnetii* conferred a significant protection to naïve recipient mice. This suggests that the surface-exposed proteins of *C. burnetii* could activate CD4^+^ T cells to protect against *C. burnetii* infection. Therefore, the identification of epitopes in the *C. burnetii* antigens recognized by CD4^+^ T cells could help design molecular vaccines against Q fever.

In the present study, we focused on the epitopes of high-abundance membrane-associated MIPs from *C. burnetii* that were identified previously [8], [27] including Com1, GroEL, P1, OmpA, OmpH, Mip, and YbgF. We selected 131 candidate peptides that were predicted to having a high-affinity binding capacity for the MHC class II molecule H2 I-A^b^ based on bioinformatic analyses.

Earlier studies demonstrated that IFN-γ was critical for the clearance of *C. burnetii* since it could stimulate the production of nitric oxide and reactive oxygen species (ROS) in macrophages (host cells) [14], [28]. Therefore, we used an IFN-γ ELISPOT assay to scan the epitope peptides recognized by IFN-γ-secreting CD4^+^ T cells isolated from mice immunized with parental proteins. As a result, 34 peptides that elicited strong positive responses in CD4^+^ T cells were identified in the initial screening, from which 22 peptides with distinct epitopes were selected for further analysis. Finally, 7 of these 22 peptides could induce IFN-γ production and proliferation in coxiella-specific memory CD4^+^ T cells.

Immunization with the peptides Com1_42–56_ and Mip_159–173_, and pooled 7 peptides containing the two peptides induced specific antibodies against the respective peptides. This suggests that the two peptides could contain B cell epitopes that could elicit specific antibody production. The prediction of B cell epitopes using IEDB analysis tools suggested that Mip_161–167_, OmpA_152–157_, and YbgF_187–194_ belong to the linear B cell epitopes of Mip, OmpA, and YbgF, respectively. However, no antibodies against *C. burnetii* or any of the MIPs used in this study were detectable in immune sera, consistent with a previous study [8]. We hypothesize that the linear epitopes of peptides might not be recognized by antibodies in immune sera that were generated against the conformational epitopes of *C. burnetii* WCA or recombinant proteins.

IL-12p70 is one of the most important cytokines for the development of Th1 cells and the initiation of cell-mediated immune responses. IL-2, IFN-γ, and TNF-α are the main cytokines secreted by Th1 cells and their secretion correlates with protection against intracellular pathogens [29], [30]. In contrast, IL-4 is the main cytokine secreted by Th2 cells, which promotes B cell proliferation and antibody generation [31]. Significantly higher levels of IL-2, IL-12p70, IFN-γ, and TNF-α were detected in the serum of mice immunized with the pooled 7 peptides, suggesting that peptide pool could efficiently activate T cells and drive their differentiation towards the Th1-polarizing phenotype.

To further characterize the CD4^+^ T cell response induced by these peptides, the intracellular expression of IFN-γ and TNF-α were measured. Compared with the negative control, 3 individual peptides from GroEL, OmpH, and YbgF stimulated the significant higher production of both IFN-γ and TNF-α, although the levels were significantly lower than those induced by *C. burnetii* WCA. Interestingly, the pool of the 7 peptides induced significantly higher expression of both IFN-γ and TNF-α in CD4^+^ T cells compared with any peptide individually, consistent with a previous study [8]. The expression of IFN-γ in CD4^+^ T cells induced by the peptide pool was comparable to that induced by *C. burnetii* WCA, whereas the expression of TNF-α induced by the peptide pool was only one third of that induced by WCA.

Finally, each of these 7 peptides and the pool of these peptides were respectively used to immunize mice to evaluate their protective efficacies against *C. burnetii* infection. Day 7 after challenge of *C. burnetii* was chosen as the time points for detection of *C. burnetii* loads and measurement of splenomegaly because the highest level of *C. burnetii* in spleens of the infected mice was found at this time and then gradually decreased in previous studies [16], [32]. Our results showed that *C. burnetii* loads and spleen weights of mice immunized with the pool of 7 peptides, but not the individual peptides, were significantly lower than negative controls. This suggests that these Th1 peptides could work in combination to efficiently activate CD4^+^ T cells and produce the Th1-type immune response against *C. burnetii* infection, but further studies are required to test whether the peptides pool could confer protection against *C. burnetii* challenge at different time points. In addition, the peptide pool could not confer complete protection, unlike *C. burnetii* WCA. Therefore, novel epitopes from other protein antigens of *C. burnetii* and additional epitope combinations should be investigated in future studies to enhance Th1 immune responses, such as promote TNF-α production of CD4^+^ T cells.

In the present study, we demonstrated that a peptide pool including Th1 epitopes of MIPs of *C. burnetii* could efficiently induce Th1 immune responses to regulate *C. burnetii* infection. These results can enhance our understanding of the mechanisms of antigen-specific CD4^+^ T cell immunity induced by *C. burnetii* epitopes, and contribute to the rational design of molecular vaccines against Q fever.

## Supporting Information

Table S1 **ELISPOT screening and MTT assay of the predicted epitope peptides.**
(DOC)
