# Supplementary material for: Exploratory Study on Th1 Epitope-Induced Protective Immunity against Coxiella burnetii Infection
Source: PLoS One. 2014 Jan 30;9(1):e87206. doi: 10.1371/journal.pone.0087206 (PMC3907486; doi:10.1371/journal.pone.0087206)
Supplement: Table S1 — ELISPOT screening and MTT assay of the predicted epitope peptides. (DOC) [file pone.0087206.s001.doc]

**Table S1. ELISPOT screening and MTT assay of the predicted epitope peptides**

| **Peptide ID** | **Sequence** | **SI^a^-rAgs^b^** | **SI^a^-WCA**  **vaccination^c^** | **CD4^+^ T cell proliferation^d^** |
| --- | --- | --- | --- | --- |
|
| Com1_42-56_ | HYLVNHPEVLVEASQ | 9.17±2.08 | 24.83±1.53^*^ | 1.34±0.06^*^ |
| Com1_80-94_ | KLFNDPASPVAGNPH | 2.78±0.94 | 3.67±0.58 | 1.1±0.03 |
| GroEL_15-29_ | AMSRGVEVLANAVKV | 1.36±0.17 |  |  |
| GroEL_16-30_ | MSRGVEVLANAVKVT | 1.34±0.31 |  |  |
| GroEL_17-31_ | SRGVEVLANAVKVTL | 1.82±0.43 |  |  |
| GroEL_37-51_ | NVVLDKSFGAPTITK | 1.64±0.18 |  |  |
| GroEL_38-52_ | VVLDKSFGAPTITKD | 1.85±0.67 |  |  |
| GroEL_39-53_ | VLDKSFGAPTITKDG | 1.84±0.51 |  |  |
| GroEL_62-76_ | LEDKFENMGAQMVKE | 2.35±0.39 | 3.33±1.41 | 1.09±0.06 |
| GroEL_63-77_ | EDKFENMGAQMVKEV | 2.02±1.07 |  |  |
| GroEL_64-78_ | DKFENMGAQMVKEVA | 1.57±0.06 |  |  |
| GroEL_365-379_ | LQERLAKLAGGVAVI | 2.50±0.80 | 3.67±1.15 | 1.19±0.12 |
| GroEL_366-380_ | QERLAKLAGGVAVIK | 1.96±0.29 |  |  |
| GroEL_367-381_ | ERLAKLAGGVAVIKV | 2.20±0.41 |  |  |
| GroEL_368-382_ | RLAKLAGGVAVIKVG | 1.60±0.40 |  |  |
| GroEL_441-455_ | EIARRAMAYPLSQIV | 1.60±0.36 |  |  |
| GroEL_442-456_ | IARRAMAYPLSQIVK | 2.20±0.52 |  |  |
| GroEL_443-457_ | ARRAMAYPLSQIVKN | 2.31±0.38 | 3.67±0.58 | 1.15±0.12 |
| GroEL_452-466_ | SQIVKNTGVQAAVVA | 1.18±0.11 |  |  |
| GroEL_453-467_ | QIVKNTGVQAAVVAD | 1.66±0.48 |  |  |
| GroEL_471-485_ | NHKDVNYGYNAATGE | 1.55±0.12 |  |  |
| GroEL_472-486_ | HKDVNYGYNAATGEY | 3.14±0.51 |  |  |
| GroEL_473-487_ | KDVNYGYNAATGEYG | 4.37±1.22 |  |  |
| GroEL_474-488_ | DVNYGYNAATGEYGD | 6.25±0.36 | 24.62±2.13^*^ | 1.27±0.08^*^ |
| GroEL_498-512_ | TKVTRTALQNAASIA | 1.43±0.33 |  |  |
| GroEL_499-513_ | KVTRTALQNAASIAG | 1.98±0.15 |  |  |
| GroEL_500-514_ | VTRTALQNAASIAGL | 1.84±0.62 |  |  |
| GroEL_501-515_ | TRTALQNAASIAGLM | 1.93±0.42 |  |  |
| Mip_12-26_ | GLLLGTTAHAATPLK | 1.90±0.21 |  |  |
| Mip_13-27_ | LLLGTTAHAATPLKT | 1.99±0.13 |  |  |
| Mip_14-28_ | LLGTTAHAATPLKTE | 1.77±0.14 |  |  |
| Mip_128-142_ | YKVLQAGQGQSPTLN | 2.04±0.09 | 1.13±0.75 | 1.13±0.09 |
| Mip_129-143_ | KVLQAGQGQSPTLND | 1.85±0.17 |  |  |
| Mip_130-144_ | VLQAGQGQSPTLNDE | 1.78±0.25 |  |  |
| Mip_159-173_ | FDSSYKRGQPATFPL | 2.10±0.13 | 12.41±0.71^*^ | 1.32±0.01^*^ |
| Mip_161-175_ | SSYKRGQPATFPLKS | 1.98±0.05 |  |  |
| Mip_190-204_ | GAIWEIYVPPQLAYG | 2.27±0.83 | 2.00±0.78 | 1.18±0.01 |
| Mip_191-205_ | AIWEIYVPPQLAYGE | 2.33±0.37 |  |  |
| Mip_197-211_ | VPPQLAYGEQGAPGV | 1.92±0.25 |  |  |
| Mip_199-213_ | PQLAYGEQGAPGVIG | 1.95±0.13 |  |  |
| Mip_200-214_ | QLAYGEQGAPGVIGP | 1.92±0.13 |  |  |
| Mip_201-215_ | LAYGEQGAPGVIGPN | 1.94±0.12 |  |  |
| Mip_202-216_ | AYGEQGAPGVIGPNE | 1.95±0.17 |  |  |
| P1_13-27_ | ALCCLASAAFAGGPD | 2.14±0.34 | 2.50±1.52 | 1.29±0.08^*^ |
| P1_15-29_ | CCLASAAFAGGPDIP | 1.74±0.07 |  |  |
| P1_18-32_ | ASAAFAGGPDIPMID | 1.95±0.20 |  |  |
| P1_41-55_ | GFGYKSYTYDQVGTV | 1.69±0.20 |  |  |
| P1_44-58_ | YKSYTYDQVGTVTVT | 1.77±0.35 |  |  |
| P1_46-60_ | SYTYDQVGTVTVTTN | 1.56±0.71 |  |  |
| P1_60-74_ | NGGTVLSVLHPVSAS | 1.77±0.05 |  |  |
| P1_62-76_ | GTVLSVLHPVSASIT | 1.66±0.53 |  |  |
| P1_65-79_ | LSVLHPVSASITQFG | 1.75±0.27 |  |  |
| P1_70-84_ | PVSASITQFGPVGEL | 2.23±0.51 | 16.00±0.71^*^ | 1.4±0.02^*^ |
| P1_83-97_ | ELGYTFASDWWIAGV | 0.59±0.17 |  |  |
| P1_86-100_ | YTFASDWWIAGVKAQ | 1.43±0.08 |  |  |
| P1_89-103_ | ASDWWIAGVKAQYQY | 1.55±0.47 |  |  |
| P1_106-120_ | VRSVHIMDAPLVGSN | 1.63±0.66 |  |  |
| P1_108-122_ | SVHIMDAPLVGSNYS | 1.60±0.11 |  |  |
| P1_111-125_ | IMDAPLVGSNYSYRT | 1.74±0.21 |  |  |
| P1_130-344_ | HLTAMLLAGIKVNEA | 1.08±0.18 |  |  |
| P1_136-150_ | LAGIKVNEANAVYLE | 1.60±0.10 |  |  |
| P1_145-159_ | NAVYLEAGYSTVWGK | 1.37±0.30 |  |  |
| P1_157-171_ | WGKTTLFGPGPVAVS | 1.54±0.12 |  |  |
| P1_159-173_ | KTTLFGPGPVAVSMK | 1.74±0.41 |  |  |
| P1_161-175_ | TLFGPGPVAVSMKNR | 1.62±0.25 |  |  |
| P1_196-210_ | DLSYDYALYRSKSNS | 2.49±0.53 | 1.52±1.38 | 1.18±0.01 |
| P1_201-215_ | YALYRSKSNSVTLSS | 1.58±0.32 |  |  |
| P1_204-218_ | YRSKSNSVTLSSATA | 2.07±0.05 | 1.63±1.08 | 1.25±0.08 |
| P1_206-220_ | SKSNSVTLSSATASA | 1.76±0.41 |  |  |
| P1_209-223_ | NSVTLSSATASAEGT | 1.58±0.04 |  |  |
| P1_226-240_ | GVSGTVQNPKRVAIN | 1.84±0.06 |  |  |
| P1_235-249_ | KRVAINGITATVNYL | 1.08±0.28 |  |  |
| OmpA_24-38_ | LAGGPDYVPAPSYAG | 1.77±0.75 |  |  |
| OmpA_25-39_ | AGGPDYVPAPSYAGV | 1.17±0.34 |  |  |
| OmpA_26-40_ | GGPDYVPAPSYAGVY | 2.49±0.65 | 1.62±0.78 | 1.09±0.05 |
| OmpA_27-41_ | GPDYVPAPSYAGVYL | 1.03±0.32 |  |  |
| OmpA_146-160_ | GKLGVAYTYNRANAG | 2.78±0.50 | 4.00±2.00^*^ | 1.29±0.03^*^ |
| OmpA_147-161_ | KLGVAYTYNRANAGL | 2.74±0.29 |  |  |
| OmpA_148-162_ | LGVAYTYNRANAGLP | 1.84±0.27 |  |  |
| OmpA_149-163_ | GVAYTYNRANAGLPT | 1.80±0.76 |  |  |
| OmpA_151-165_ | AYTYNRANAGLPTNK | 1.75±0.72 |  |  |
| OmpA_152-166_ | YTYNRANAGLPTNKI | 1.59±0.40 |  |  |
| OmpA_171-185_ | GSRSRFWNPLFAAGV | 0.98±0.43 |  |  |
| OmpA_172-186_ | SRSRFWNPLFAAGVQ | 1.00±0.60 |  |  |
| OmpA_173-187_ | RSRFWNPLFAAGVQY | 1.61±0.28 |  |  |
| OmpA_197-211_ | AQYTFVPGYRNASSK | 2.70±0.33 | 2.25±0.65 | 1.21±0.03 |
| OmpA_198-212_ | QYTFVPGYRNASSKR | 2.37±0.20 |  |  |
| OmpA_207-221_ | NASSKRFVAPVTHLF | 1.27±0.28 |  |  |
| OmpA_208-222_ | ASSKRFVAPVTHLFT | 1.59±0.31 |  |  |
| OmpH_9-23_ | ICLSVAMIWSVAAVA | 2.00±1.09 |  |  |
| OmpH_10-24_ | CLSVAMIWSVAAVAQ | 2.81±1.74 | 2.75±0.78 | 1.28±0.07^*^ |
| OmpH_11-25_ | LSVAMIWSVAAVAQT | 2.71±0.81 |  |  |
| OmpH_12-26_ | SVAMIWSVAAVAQTV | 2.12±1.74 |  |  |
| OmpH_13-27_ | VAMIWSVAAVAQTVG | 2.82±0.76 | 4.00±1.73^*^ | 1.4±0.04^*^ |
| OmpH_14-28_ | AMIWSVAAVAQTVGL | 1.95±1.33 |  |  |
| OmpH_15-29_ | MIWSVAAVAQTVGLV | 1.14±0.08 |  |  |
| OmpH_16-30_ | IWSVAAVAQTVGLVD | 2.28±0.91 | 1.5±1.13 | 1.37±0.03^*^ |
| OmpH_17-31_ | WSVAAVAQTVGLVDM | 1.82±0.39 |  |  |
| OmpH_18-32_ | SVAAVAQTVGLVDMR | 1.73±1.18 |  |  |
| OmpH_28-42_ | LVDMRQIFQTAPQIK | 1.88±0.28 |  |  |
| OmpH_29-43_ | VDMRQIFQTAPQIKD | 1.94±0.16 |  |  |
| OmpH_30-44_ | DMRQIFQTAPQIKDI | 1.90±0.56 |  |  |
| OmpH_31-45_ | MRQIFQTAPQIKDIN | 1.96±1.27 |  |  |
| OmpH_119-133_ | SDFMSKVNGAVKRVA | 1.83±0.25 |  |  |
| YbgF_8-22_ | IKTLCVSSALAALML | 1.85±0.14 |  |  |
| YbgF_9-23_ | KTLCVSSALAALMLS | 1.85±0.19 |  |  |
| YbgF_16-30_ | ALAALMLSAPLTWAD | 1.93±0.11 |  |  |
| YbgF_31-45_ | APVEDISAQPQPTKT | 1.83±0.25 |  |  |
| YbgF_32-46_ | PVEDISAQPQPTKTT | 1.2±0.11 |  |  |
| YbgF_33-47_ | VEDISAQPQPTKTTV | 1.13±0.26 |  |  |
| YbgF_34-48_ | EDISAQPQPTKTTVS | 2.07±0.14 | 2.08±1.67 | 1.19±0.07 |
| YbgF_50-64_ | SETPETAIPTAPVSL | 1.96±0.08 |  |  |
| YbgF_51-65_ | ETPETAIPTAPVSLP | 1.73±0.28 |  |  |
| YbgF_53-67_ | PETAIPTAPVSLPTT | 1.54±0.25 |  |  |
| YbgF_56-70_ | AIPTAPVSLPTTQTD | 2.10±0.04 | 1.50±0.65 | 1.16±0.03 |
| YbgF_185-199_ | LLTKKQYDKAQASFQ | 2.14±0.11 | 9.33±2.94^*^ | 1.27±0.08^*^ |
| YbgF_186-200_ | LTKKQYDKAQASFQN | 2.07±0.08 |  |  |
| YbgF_187-201_ | TKKQYDKAQASFQNY | 2.06±0.01 |  |  |
| YbgF_274-288_ | EIKKQHPESTAAQLA | 1.81±0.10 |  |  |
| YbgF_275-289_ | IKKQHPESTAAQLAN | 1.85±0.19 |  |  |
| YbgF_277-291_ | KQHPESTAAQLANIR | 1.87±0.20 |  |  |

^a^Stimulation index (SI) was calculated using the number of number of spot forming cells (SFC) in peptide-stimulated cells divided by the number of SFC in non-stimulated cells; SI >2 was considered to be positive.

^b^CD4^+^T cells were isolated from 5 mice immunized with 20 μg of recombinant protein on day 10 post immunization. Peptides were added at 2μg/ml for 20 h, three independent experiments.

^c^CD4^+^T cells were isolated from 5 mice immunized with 20 μg of WCA of *C. burnetii* on day 10 post immunization. Peptides were added at 2μg/ml for 20 h, three independent experiments.

^d^CD4^+^T cells were isolated from 5 mice immunized with 20 μg of WCA of *C. burnetii* on day 10 post immunization. Peptides were added at 10 μg/ml for 48 h, three independent experiments. The proliferation level of CD4^+^T cells was calculated as the absorb value of T cells stimulated with each peptide divided by that of T cells stimulated with PBS.
